# Infra-Low Frequency Neurofeedback in Tension-Type Headache: A Cross-Over Sham-Controlled Study

**DOI:** 10.3389/fnhum.2022.891323

**Published:** 2022-05-20

**Authors:** Galina A. Arina, Olga R. Dobrushina, Elizaveta T. Shvetsova, Ekaterina D. Osina, Georgy A. Meshkov, Guzel A. Aziatskaya, Alexandra K. Trofimova, Inga N. Efremova, Sergey E. Martunov, Valentina V. Nikolaeva

**Affiliations:** ^1^Faculty of Psychology, M. V. Lomonosov Moscow State University, Moscow, Russia; ^2^International Institute of Psychosomatic Health, Moscow, Russia; ^3^Research Center of Neurology, Moscow, Russia; ^4^Yandex LLC, Moscow, Russia; ^5^Federal State Budgetary Institution “Federal Center of Brain Research and Neurotechnologies” of the Federal Medical Biological Agency, Moscow, Russia

**Keywords:** primary headache, tension-type headache, endogenous neuromodulation, neurofeedback, infra-low frequency

## Abstract

Primary headaches are highly prevalent and represent a major cause of disability in young adults. Neurofeedback is increasingly used in the treatment of chronic pain; however, there are few studies investigating its efficacy in patients with headaches. We report the results of a cross-over sham-controlled study on the efficacy of neurofeedback in the prophylactic treatment of tension-type headache (TTH). Participants received ten sessions of infra-low frequency electroencephalographic neurofeedback and ten sessions of sham-neurofeedback, with the order of treatments being randomized. The study also included a basic psychotherapeutic intervention — a psychoeducational session performed before the main study phases and emotional support provided throughout the study period. The headache probability was modeled as a function of the neurofeedback and sham-neurofeedback sessions performed to date. As a result, we revealed a strong beneficial effect of neurofeedback and no influence of the sham sessions. The study supports the prophylactic use of infra-low frequency neurofeedback in patients with TTH. From a methodological point of view, we advocate for the explicit inclusion of psychotherapeutic components in neurofeedback study protocols.

## Introduction

Tension-type headache (TTH) is among the most common diseases: the percentage of the adult population with an active TTH disorder is 38% (Jensen and Stovner, 2008). Despite being benign, primary headache is among the ten most disabling disorders for both sexes and among the five most disabling disorders for women (Stovner et al., 2007), and is most burdensome in women aged 15 to 49 years (Stovner et al., 2018). Currently, pharmacotherapy remains the most established treatment for TTH, despite the substantial risk of side effects. Non-drug treatment methods, including biofeedback and neuromodulation, are promising alternatives to medications (Nestoriuc et al., 2008; Bendtsen et al., 2010; Ailani et al., 2021).

Neurofeedback is a type of biofeedback utilizing brain signals and, at the same time, is it the least interventional type of neuromodulation, that relies on brain training rather than on direct stimulation. Neurofeedback targets the therapeutic modulation of a dysfunctional brain state, such as an imbalance in electroencephalographic (EEG) activity or altered intrinsic connectivity patterns (Ros et al., 2013; Marzbani et al., 2016; Nicholson et al., 2016; Dobrushina et al., 2020). Despite the increasing number of studies examining neurofeedback in chronic pain (Roy et al., 2020), only a few have focused on patients with headache. While some studies show a reduction in migraine severity after neurofeedback (Siniatchkin et al., 2000; Stokes and Lappin, 2010; Walker, 2011), the available data on TTH are limited to the results of a single randomized study addressing primary headaches in general (Moshkani Farahani et al., 2014). This study utilized brain oscillation power-based neurofeedback — a type of neurofeedback based on the voluntary modulation of certain EEG frequencies, and a waiting list control; it demonstrated a reduction in headache frequency after neurofeedback.

In the current article, we present the results of a cross-over sham-controlled study evaluating the effects of infra-low frequency EEG neurofeedback in patients with TTH. Infra-low frequency neurofeedback targets slow brain fluctuations and is based on the principles of implicit learning: there is no goal to increase or decrease the signal intensity, but the feedback *per se* is supposed to contribute to the augmentation of internal neural models through the mechanisms of predictive coding, similar to the role of natural feedback in neural development (Friston, 2010; Othmer et al., 2013; Ioannides, 2018). Infra-low frequency EEG fluctuations (f<0.1Hz) were discovered in the 1950s by Aladjalova, who supposed that they reflect the slow regulatory processes related to brain metabolism and that they are mediated by the hypothalamus (Aladjalova, 1957). Later, slow brain dynamics received extensive attention in the context of intrinsic connectivity networks revealed by functional MRI (Buckner et al., 2013). These networks demonstrate infra-low frequency fluctuations that are correlated with infra-low EEG fluctuations (Hiltunen et al., 2014; Haufe et al., 2018).

Alterations in brain networks are considered to be an important mechanism of pain disorders (Thorp et al., 2018). The transition from episodic to chronic TTH is believed to be linked to central sensitization rather than to peripheral mechanisms (Bendtsen, 2000). Central sensitization is related to increased connectivity of the insula with the default-mode network, and also to decreased connectivity between the antinociceptive regions (Harte et al., 2018). At the same time, centralized pain syndromes are characterized by an abnormal regulation of the hypothalamic-pituitary-adrenal axis (Eller-Smith et al., 2018). Slow regulatory brain systems associated with intrinsic connectivity networks represent a potential therapeutic target in pain disorders. Infra-low frequency neurofeedback, based on slow EEG dynamics, is known to modulate intrinsic brain connectivity (Dobrushina et al., 2020). Thus, this method can be a feasible non-pharmacological alternative for the treatment of pain disorders.

In clinical practice, infra-low frequency neurofeedback is extensively used in the treatment of headaches (Othmer, 2017), but, to our knowledge, it has not previously undergone a sham-controlled evaluation in patients with TTH. A disconnection between clinical practice and research evidence exists in the field of neurofeedback and it is a common reason for critique of this method. While studies have produced mixed results (Schabus et al., 2017; Strehl et al., 2017), intense interpersonal debates are ongoing around the possible role of placebo effects in neurofeedback interventions (Fovet et al., 2017; Witte et al., 2018). Another complex methodological issue is related to the questions raised by personalized medicine (Mathur and Sutton, 2017) — for example, is it realistic to expect neurofeedback techniques to work as a “one size fits all” solution? Keeping in mind these methodological considerations, in the current study we combined group and individual case analyses and evaluated the psychological components of the neurofeedback interventions. The study protocol included a psychoeducational intervention performed before the start of the main study phases and allowed for supportive communication between the patients and the clinician during the study period.

## Materials and Methods

### Participants and Experimental Design

The study included 8 patients (Table 1). The following inclusion criteria were used:

**TABLE 1:** Characteristics of the study sample.

ID	Age	Sex	Headache type	Headache duration, years	Baseline headache frequency, monthly headache days	Medication use	Order of phases
T1	21	Female	Episodic TTH	3	11	Preventive: None Abortive: NSAID, 1-2 times a month	Neurofeedback-Sham
T2	42	Male	Chronic TTH	27	29	Preventive: Amitriptyline 25 mg a day Abortive: None	Neurofeedback-Sham
T3	26	Female	Chronic TTH	10	18	Preventive: None Abortive: NSAID and combination analgesics, 3-4 times a month	Neurofeedback-Sham
T4	30	Female	Episodic TTH	20	13	Preventive: None Abortive: NSAID, 1-2 times a month	Sham-Neurofeedback
T5	40	Female	Episodic TTH	25	14	Preventive: None Abortive: None	Sham-Neurofeedback
T6	41	Female	Episodic TTH	30	14	Preventive: None Abortive: None	Neurofeedback-Sham
T7	25	Female	Episodic TTH	10	9	Preventive: None Abortive: NSAID, 4 times a month	Sham-Neurofeedback
T8	21	Female	Chronic TTH	13	31 (every day)	Preventive: None Abortive: NSAID, 2 times a month	Sham-Neurofeedback

• Age 18 to 45 years.
• TTH diagnosed in accordance with the International Classification of Headache Disorders, 3rd edition (Headache Classification Committee of the International Headache Society (IHS), 2013).
• Absence of other neurological disorders.
• Headache frequency of at least four times a month.
• Stable life circumstances (absence of major life changes during the study period).
• In case preventive pharmacotherapy was used, it was required that it was started at least one month before enrollment and continued in a constant dose. Patients were allowed to take any abortive medications during the study.

Five patients had episodic TTH and three patients had chronic TTH. Patients T1, T3, T4, T7, T8 were using NSAID or combination analgesics when the pain significantly interfered with daily activities, while other avoided abortive therapy due to limited efficacy and the risk side effects. Patient T2, who had concurrent depression, received amitriptyline as preventive therapy; the medication improved his mood and functional state, but the headache continued on a daily basis.

The study protocol was approved by the Ethics Committee of the M. V. Lomonosov Moscow State University, and all participants gave informed consent for participation. Specifically, informed consent included a description of the infra-low frequency neurofeedback process and principles. The dataset for this study can be found in the Mendeley Repository: http://dx.doi.org/10.17632/z9s6nk6s99.1.

The study included 4 phases: (1) pre-intervention (at least 3 weeks), (2) first intervention (neurofeedback or sham-neurofeedback, 5 weeks), (3) second intervention (neurofeedback or sham-neurofeedback, 5 weeks), (4) catamnesis (at least 3 weeks). There was a 2-week break between the end of second and the start of the third phases (no intervention but continuing to fill in the diary). Each intervention phase included 10 sessions of either neurofeedback or sham-neurofeedback. The order of interventions (neurofeedback first or sham-neurofeedback first) was randomized. The patients were blinded to the randomization order.

Before the first study phase patients received a single psychoeducational session that included an explanation of TTH mechanisms, including the benign nature of the headache and the risk of medication overuse, and training in progressive muscular relaxation. Patients were given an audio recording of the relaxation technique and were encouraged to use it 3 times a week. They were allowed to contact the clinician in case they had any questions, and the clinician was instructed to give emotional support (active listening and validation).

### Neurofeedback Procedure

The infra-low frequency neurofeedback procedure was performed in accordance with the Othmer protocol (Othmer, 2017). Electroencephalographic signals were recorded with the NeuroAmp (Corscience GmbH) DC-amplifier, sampled with 1K samples per second, filtered and down-sampled to 250 samples per second and 32-bit resolution, from Ag/AgCl sintered electrodes. Skin was prepared with NuPrep abrasive paste, and the electrodes were fixed with Ten-20 conductive paste to reach an impedance below 5 kOhm. During the sham phase, a simulated signal was created by a random number generator, and the spectral power density was shaped to match that of a typical EEG. The electrodes were positioned at the P4 and T4 sites (10–20 system). The reference electrode was placed at Cz and the ground electrode was placed on the forehead. The clinician asked patients about their sensations during the sessions and, in the neurofeedback phase, they adjusted the reward frequency in case of any signs of sedation or excitation, in accordance with the infra-low frequency neurofeedback approach (Othmer, 2017).

Cygnet biofeedback software (BEE Medic GmbH) was used for signal processing and feedback imagery presentation, with either the Inner Tube or Dreamscapes neurofeedback games (Somatic Vision Inc.). In the Inner Tube game the patients watched a rocket moving through tunnels, while in the Dreamscapes game the patients “moved” through an artificial landscape. The speed of the movement and, in the Dreamscapes game, the brightness of the image were governed in real time by the infra-low frequency band-limited waveform of the EEG signal. Due to the implicit principle of infra-low frequency neurofeedback, there was no goal to voluntarily influence the speed or brightness, and the participants were instructed to watch the visualization without trying to control it.

### Assessment Methods

At enrollment, the study participants completed a set of questionnaires — the McGill Pain Questionnaire (MPQ) (Melzack, 1975), the Beck Depression Inventory (BDI) (Beck et al., 1961), the Spielberger State-Trait Anxiety Inventory (STAI) (Spielberger et al., 1983) and the Minnesota Multiphasic Personality Inventory (MMPI) (Hathaway and McKinley, 1943). Patients with TTH were also assessed for alexithymia with the use of the Toronto Alexithymia Scale 20 (TAS-20) (Bagby et al., 1994).

During the study period, including the pre-intervention and catamnestic phases, the participants were required to fill in a headache diary that included information on the duration and intensity of headaches and medication use. Since the use of abortive medications, which affect headache intensity and duration, was allowed, only the presence or absence of headache was used as an outcome variable. The total numbers of observed days for all patients were 1119.

After the study period, all the patients excluding T2 were interviewed about their beliefs regarding the mechanisms of neurofeedback, their *a priori* expectations regarding the efficacy of neurofeedback, their behavior during the session and their beliefs about their owns role in the neurofeedback.

### Group Data Analysis

Data analysis was performed with the use of mixed linear modeling in R Project 3.3.3,^1^ packages “lme4,” “lmerTest,” “simr,” “reshape2,” “dplyr,” “ggplot2,” and “directlabels.”

As an *a priori* assumption, we considered the effect of neurofeedback to be cumulative. Thus, the dependency of the probability of a subject to have a headache in a given day was modeled as a function of the total count of neurofeedback and the total count of sham sessions the participant had to date. We used a generalized linear mixed effects model with binomial distribution (logit link) for the dependent variable *hadPain* (having a headache on a given day), entering the *toDateNeurofeedback* (count of neurofeedback sessions to date) and *toDateSham* (count of sham sessions to date) variables as fixed effects. The random effects related to individual patients — *subject* variable (subject id), were applied both to the intercept and to the slopes. The final generalized linear mixed effects model formula was as follows:


hadPain∼toDateNeurofeedback+toDateSham+(1+toDateNeurofeedback+toDateSham|subject)

The patient T8 had a headache every day, making the modeling of headache frequency non-informative (the model would not converge). The evaluation of daily pain duration in this patient indicated that she most frequently had 7 or 10 h of pain a day, with 65% of days having more than 7 pain hours a day and 30% of days having more than 10 pain hours a day. Thus, we decided to classify participant T8 as having a headache on a given day (hadPain = 1) only when the pain duration exceeded 7 h.

For the power estimation, we used the data from the study by Stokes and Lappin (2010), indicating a reduction in the odds of having a headache on a given day from 0.34 at baseline to 0.11 after the course of neurofeedback treatment (0.11/0.34 = 0.31 of the initial odds). Such a reduction in odds corresponds to the logistic regression slope for *toDateNeurofeedback* of –0.117: exp(–0.117)^10^ = 0.31. Monte Carlo simulations were used to estimate the power of the study (Green and Macleod, 2016). For the effect of *toDateNeurofeedback* of –0.117 and the zero effect of placebo, the power was estimated as 99.0% (95% confidence interval: 94.6–99.9%). For the smaller effect size that we factually observed in our study, i.e., –0.088 (see section “Group efficacy of neurofeedback” below), the power estimate was 97.0% (95% confidence interval: 91.5–99.4%).

### Individual Case Data Analysis

For the individual case analysis, the probability of each subject to have a headache on a given day was modeled as a function of the total counts of neurofeedback and sham sessions the participant had to date, using the generalized linear model (binomial distribution):


hadPain∼toDateNeurofeedback+toDateSham

Based on the logistic regression slope for *toDateNeurofeedback*, the participants were classified as neurofeedback responders or non-responders. A subsequent qualitative analysis of the patient’s baseline characteristics was performed with the aim of identifying the features differentiating responders and non-responders.

## Results

### Group Efficacy of Neurofeedback

**FIGURE 1:**
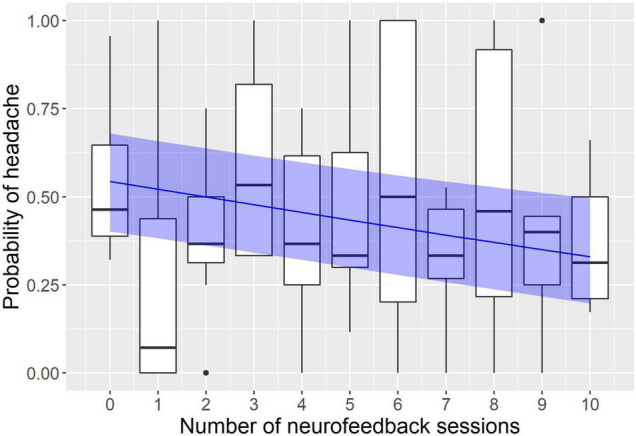
Modeling of the influence of neurofeedback on the headache probability. The x axis represents the number of neurofeedback sessions the subject had to date; the y axis represents the probability to have a headache on a given day. Observed probabilities are shown by boxplots in black (the lower and upper hinges correspond to the first and third quartiles; the bold horizontal line corresponds to the median; the whiskers extend to the largest value no further than 1.5 inter-quartile range from the hinge; outliers are shown by dots). The probability predicted by the model is indicated by the blue line with a ribbon representing 95% confidence interval.

**FIGURE 2:**
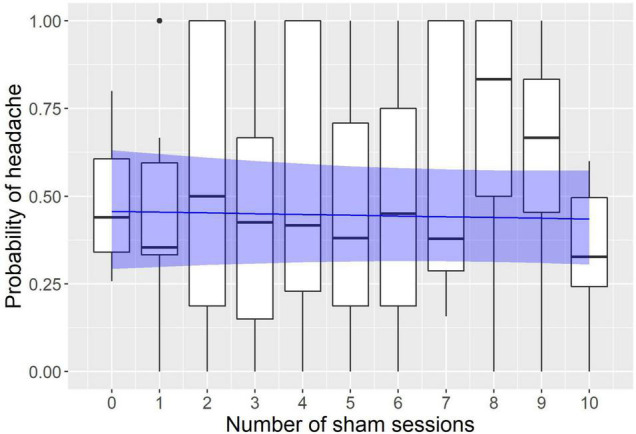
Modeling of the influence of sham sessions on the headache probability. The x axis represents the number of sham sessions the subject had to date; the y axis represents the probability to have a headache on a given day. Observed probabilities are shown by boxplots in black (the lower and upper hinges correspond to the first and third quartiles; the bold horizontal line corresponds to the median; the whiskers extend to the largest value no further than 1.5 inter-quartile range from the hinge; outliers are shown by dots). The probability predicted by the model is indicated by the blue line with a ribbon representing 95% confidence interval.

The modeling revealed a significant effect of neurofeedback and no effect of sham sessions (see Figures 1, 2). The logistic regression slope for the number of neurofeedback sessions performed to date was -0.088 ± 0.026 (*p* = 0.0007), and for the number of sham sessions, the slope was -0.009 ± 0.031 (*p* = 0.77). This means that after each neurofeedback session, the odds of having TTH on subsequent days changed by the ratio of exp(-0.088) = 0.92, i.e., it decreased by 8%. For the 10 sessions, it would be 0.92^10^ = 0.43, i.e., almost half the odds before treatment.

### Individual Case Analysis

The individual case analysis revealed a reduction in headache frequency related to neurofeedback in patients T1, T2, T5, T6, T7, and T8; the effect reached the level of statistical significance in patients T1, T6, and T7 (Table 2). The effect size for the observed slope of less than -0.1 corresponds to the reduction in the odds of having a headache to 37% from the baseline odds after 10 neurofeedback sessions [exp(-0.1)^10^ = 0.37]. Thus, we considered the size of the effect in patients T1, T2, T5, T6, T7, and T8 to be of clinical significance and classified them as neurofeedback responders. In patients T3 and T4, no effects of neurofeedback were observed; they were classified as non-responders. Sham neurofeedback resulted in improvement in patient T8 (placebo effect), while in patients T6 and T7 it led to an exacerbation of their headaches (nocebo effect).

**TABLE 2:** Results of the general linear modeling in individual cases for the tension-type headache study.

Patient	NF slope	NF *p*-value	Sham slope	Sham *p*-value
T1	–0.126	0.019	–0.012	0.835
T2	–0.105	0.117	–0.014	0.823
T3	–0.045	0.243	–0.003	0.945
T4	0.004	0.944	–0.065	0.235
T5	–0.193	0.105	–0.028	0.595
T6	–0.260	0.001	0.208	0.025
T7	–0.121	0.041	0.114	0.037
T8	–0.129	0.128	–0.150	0.049

**FIGURE 3:**
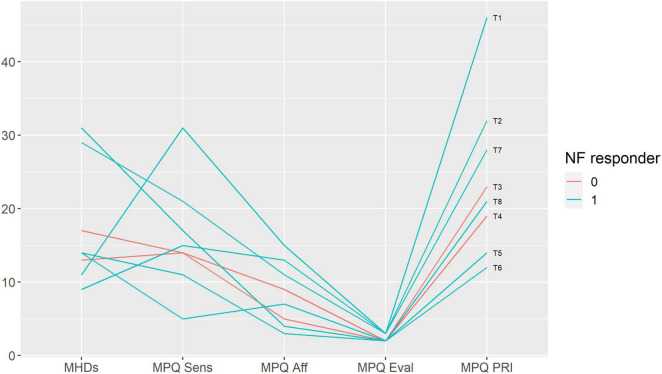
Baseline pain characteristics in participants with and without response to neurofeedback. Each line represents a patient case (indicated on the right end of the line). Neurofeedback responders are show in blue, neurofeedback non-responders — in red color. MHDs — headache frequency, monthly headache days; MPQ — McGill Pain Questionnaire: Sensory (Sens), Affective (Aff), Evaluative (Eval) scores and the Pain Rating Index (PRI); NF — neurofeedback.

**FIGURE 4:**
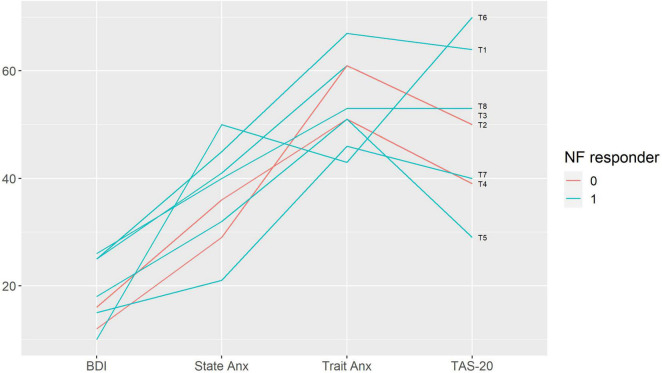
Baseline emotional characteristics in participants with and without response to neurofeedback. Each line represents a patient case (indicated on the right end of the line). Neurofeedback responders are show in blue, neurofeedback non-responders — in red color. BDI — Beck Depression Inventory; State Anx — State Anxiety, Trait Anx — Trait Anxiety (Spielberger State-Trait Anxiety Inventory); TAS-20 — Toronto Alexithymia Scale 20; NF — neurofeedback.

**FIGURE 5:**
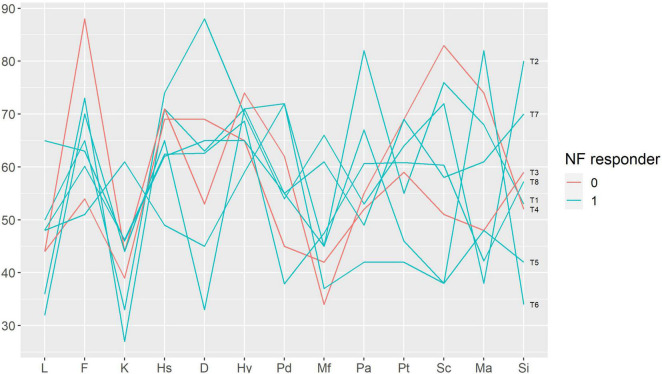
Baseline Minnesota Multiphasic Personality Inventory profiles in participants with and without response to neurofeedback. Each line represents a patient case (indicated on the right end of the line). Neurofeedback responders are show in blue, neurofeedback non-responders — in red color. Minnesota Multiphasic Personality Inventory (MMPI) scales: L, F, K — validity scales, Hs — hypochondriasis, D — depression, Hy — hysteria, Pd — psychopathic deviate, Mf — masculinity-femininity, Pa — paranoia, Pt — psychasthenia, Sc — schizophrenia, Ma — mania, Si — social introversion; NF — neurofeedback.

We performed a qualitative analysis in order to search for the baseline characteristics discriminating potential neurofeedback responders and non-responders. The non-responders did not differ from the rest of the sample in terms of pain characteristics (Figure 3); anxiety, depression and alexithymia (Figure 4); and personality traits (Figure 5). Post-study interviews showed that, despite reading the same informed consent information, the participants developed very different and sometimes bizarre explanations of the infra-low neurofeedback mechanisms, based on their experience (Table 3). Two participants had realistic neurophysiological explanations, three participants thought that neurofeedback is related to some physiological signal such as breath or blood flow, one participant though that neurofeedback is like hypnosis and another one did not reflect about the neurofeedback principles. At the start on the study, four participants had positive expectations about neurofeedback efficacy, two — mixed, and one — negative expectations. Despite being given the instruction to “just watch,” five participants tried to control the feedback game and sometimes reached conclusions about their influence on the feedback parameters that are unexplainable from the neurophysiological point of view. The beliefs about neurofeedback and behavior during the sessions seemed to have no influence on the neurofeedback’s efficacy. Regarding the patient’s *a priori* expectations about the efficacy of neurofeedback, an initial skeptical attitude did not preclude neurofeedback-related improvements, while a strong belief in the method did not prevent the intervention from being ineffective.

**TABLE 3:** Results of the post-study interviews.

Patient	Beliefs regarding the mechanisms of neurofeedback	*A priori* expectations about the efficacy of neurofeedback	Behavior during the session and beliefs about one’s own role in the neurofeedback
T1 responder	Did not reflect about the principles of neurofeedback.	Was interested to see how neurofeedback would work; expected to see positive results.	Experienced strong relaxation; had to think intensively in order not to fall asleep.
T3 non-responder	Thought that the neurofeedback imagery induced relaxation, may be like hypnosis.	Hoped very much that the method would help, since she had strong pain and nothing helped.	Tried to find how the image was related to her inner feelings; was unable to find anything but believed that the rocket depended on her state.
T4 non-responder	Thought that the speed of the rocket was linked to some physiological processes in her body.	At the beginning of the study, had a strong belief that the neurofeedback would help. In the middle of the study, had doubts about the efficacy of the method and experienced low motivation to come to the trainings [sham phase first]. At the end of the study, the doubts disappeared.	Tried to find how she can influence the neurofeedback game but failed. At the beginning of the study, tried to concentrate on the training, but instead got into a relaxed state. Afterward, just relaxed.
T5 responder	Reported that she understood the mechanisms of neurofeedback after reading the informed consent: the electrodes recorded information about brain wave frequencies, and the speed of the image was influenced by the frequency of the brain.	Before the study, already heard about different biofeedback methods, and was skeptical regarding the potential of neurofeedback to influence headache. Reported that afterward, she had to accept the opposite, since she noticed improvements in headache during and after the study.	Thought that she could influence the speed of the rocket and tried to make experiments. When the rocket was flying too fast, felt drowsiness and tried to slow the rocket down by moving the gaze away (following the rocket with side view), which she believed made the speed more comfortable.
T6 responder	Electrodes registered information about the brain waves, and the image was changing in accordance with brain waves. “I looked at this image and by a cycled mechanism the processes in neural chains influenced what I saw.”	Had a strong hope that neurofeedback would help (because authoritative people told her about this method), but at the same time was skeptical (“could not imagine that it would help”). After the second training, noticed improvement in headache and gained a strong belief that neurofeedback is helpful [neurofeedback phase first]. Closer to the end of the study, was unsure if neurofeedback could help her (experienced stress related to the pandemic and an increase in headache frequency).	Had no feeling that she could consciously influence something. Decided that the speed of the rocket was influenced by her state: when she was active, the rocket was faster; when she was tired, it was slower.
T7 responder	Thought that the speed of the rocket was influenced by the blood flow in her head (when her artery was squeezed, the rocket was moving more erratically).	Was ready to believe in anything. Had doubts regarding the potential efficacy, but was open to everything, and had a hope that neurofeedback would help.	During the second but not the first phase of the treatment, noticed that when she moved the head, the screen got dark [sham first]. Tried to speed up the rocket with her thoughts, reported it did not work.
T8 responder	Thought that the neurofeedback influences her breath, making it more relaxed and leading to general relaxation.	Before the study, thought that the neurofeedback would help her. Closer to the end of the study, had an emotional breakdown caused by an external situation – at this point, she lost her hope in the method.	Thought that she influenced the speed of the rocket, although was not understanding how it was achieved.

## Discussion

In the current study we observed clearly different responses to neurofeedback and sham-neurofeedback. While neurofeedback resulted in a marked reduction of TTH frequency, the sham intervention, as shown by the group analysis, was not associated with improvements, and the individual case analysis indicated that it could exhibit both placebo and nocebo effects. Despite the relatively low number of participants, the study was sufficiently powered due to the cross-over design and the long observation period. The results of the study are in accordance with the positive clinical experience (Othmer, 2017). As a secondary goal, we aimed to describe the individual factors potentially influencing the efficacy of neurofeedback. Based on the available data, which were limited by the low number of cases and especially of non-responders, we were unable to draw any conclusions. Surprisingly, even patients with signs of affective disorders and altered personality profiles responded to neurofeedback. At the same time, we observed that skeptical expectations and unrealistic beliefs regarding neurofeedback seemed to have no influence on its efficacy.

The choice of neurofeedback protocol — infra-low frequency neurofeedback with electrode placement at the T4P4 site (right middle temporal gyrus and the right inferior parietal lobule) — was based on previous clinical experience (Othmer, 2017). Neurofeedback from the T4P4 site is supposed to increase awareness toward incoming interoceptive and exteroceptive sensory information, to induce a mindful state. The positive experience with T4P4 infra-low frequency neurofeedback in TTH is consistent with evidence on the efficacy of mindfulness in primary headaches (Gu et al., 2018). Previously, we showed that a single infra-low frequency neurofeedback session with T4P4 placement results in increased connectivity within the areas in the right anterior insula, in the left and right rostral prefrontal cortex and in the cortex processing verbal and visual information (Dobrushina et al., 2020). These findings indicate functional integration between the multisensory areas and the high-order regulatory cortex, they are in accordance with behavioral observations. Based on the limited existing data, we can speculate that the modulation of intrinsic networks by neurofeedback may compensate for some of the alterations related to chronic pain. The increased connectivity between the insula and the default-mode network that is observed in chronic pain is believed to reflect hyper-awareness to pain — a narrowed pain-guided focus of attention (Napadow et al., 2010, 2012). Infra-low frequency neurofeedback from the T4P4 site can rebalance attention into a wider, more integrated and regulated state by the means of brain connectivity modulation.

The study protocol included not only neurofeedback, but also a basic psychotherapeutic intervention: psychoeducation and emotional support. Integrative care with a preference for non-drug interventions is supposed to be the optimal approach for the treatment of chronic headache (Bendtsen et al., 2010; Gaul et al., 2011; Wallasch and Kropp, 2012). Our clinical experience shows that infra-low frequency neuromodulation can be successfully combined with medications due to the lack of undesired interactions, and that it supports the efficacy of psychotherapy by providing a stable physiological basis. The same time, attempting to treat a chronic pain condition with any single intervention is often self-defeating. Thus, relying on the integrative approach, we allowed participants of the study to receive prophylactic medications, consulted them on abortive medications to prevent overuse and provided basic psychotherapeutic care.

Methodologically, the inclusion of the psychotherapeutic components into the study protocol can be more accurate than attempting to perform a purely neurophysiological intervention and considering non-physiological factors as artifacts. The psychoeducational session was performed 3 weeks before the start of the neurofeedback or sham-neurofeedback. Thus, the main study phases were rather “clean” from the non-specific effects related to the formation of a psychotherapeutic alliance and to the educational influence of the talks between the patient and clinician accompanying the neurofeedback/sham sessions. During neurofeedback procedures, the clinician not only connects the electrodes to the patient’s head but also spends hours with the patient, talking or being present in silence. Neurofeedback has aspects in common with psychotherapy, since it includes the development of the therapeutic milieu (Strehl, 2014), with the inevitable role of an alliance. The alliance is believed to be more than a nonspecific factor, and it has been shown to have neurobiological underpinnings (Zilcha-Mano et al., 2019). Multiple studies have demonstrated that the quality of an alliance mediates the outcome of not only psychotherapeutic but also pharmacological treatments (Krupnick et al., 2006; Cohen et al., 2017), and it is reasonable to suggest a similar role in neurofeedback. When not specified explicitly, psychotherapeutic components can add uncontrolled effects to the outcome variables, leading to false positive or false negative results.

In conclusion, the study findings provide support for the use of infra-low frequency neurofeedback in the integrative treatment of patients with TTH. To our knowledge, this is the first sham-controlled study of neurofeedback in primary headache.

## Data Availability Statement

The datasets presented in this study can be found in online repositories. The names of the repository/repositories and accession number(s) can be found below: Mendeley Repository, http://dx.doi.org/10.17632/z9s6nk6s99.1.

## Ethics Statement

The studies involving human participants were reviewed and approved by Ethics Committee of the M. V. Lomonosov Moscow State University. The patients/participants provided their written informed consent to participate in this study.

## Author Contributions

GAr, OD, and VN designed and coordinated the study. ES, EO, GAz, AT, IE, and SM collected the data. GM, GAr, and OD analyzed the data. OD, GAr, and GM wrote the manuscript. All authors contributed to the article and approved the submitted version.

## Conflict of Interest

GM is employed by Yandex LLC. The remaining authors declare that the research was conducted in the absence of any commercial or financial relationships that could be construed as a potential conflict of interest.

## Publisher’s Note

All claims expressed in this article are solely those of the authors and do not necessarily represent those of their affiliated organizations, or those of the publisher, the editors and the reviewers. Any product that may be evaluated in this article, or claim that may be made by its manufacturer, is not guaranteed or endorsed by the publisher.

## Footnotes

1 https://www.R-project.org/, RRID:SCR_001905.

## References

[B1] Ailani J. Burch R. C. Robbins M. S. (2021). The American Headache Society Consensus Statement: update on integrating new migraine treatments into clinical practice. *Headache J. Head Face Pain* 61 1021–1039. 10.1111/head.14153 34160823

[B2] Aladjalova N. A. (1957). Infra-Slow rhythmic oscillations of the steady potential of the cerebral cortex. *Nature* 179 957–959. 10.1038/179957a0 13430746

[B3] Bagby R. M. Parker J. D. A. Taylor G. J. (1994). The twenty-item Toronto Alexithymia scale—I. Item selection and cross-validation of the factor structure. *J. Psychosom. Res.* 38 23–32. 10.1016/0022-3999(94)90005-1 8126686

[B4] Beck A. T. Ward C. H. Mendelson M. Mock J. Erbaugh J. (1961). An inventory for measuring depression. *Arch. Gen. Psychiatry* 4 561–571. 10.1001/archpsyc.1961.01710120031004 13688369

[B5] Bendtsen L. (2000). Central sensitization in tension-type headache—possible pathophysiological mechanisms. *Cephalalgia* 20 486–508. 10.1046/j.1468-2982.2000.00070.x 11037746

[B6] Bendtsen L. Evers S. Linde M. Mitsikostas D. D. Sandrini G. Schoenen J. (2010). EFNS guideline on the treatment of tension-type headache - Report of an EFNS task force. *Eur. J. Neurol.* 17 1318–1325. 10.1111/j.1468-1331.2010.03070.x 20482606

[B7] Buckner R. L. Krienen F. M. Yeo B. T. T. (2013). Opportunities and limitations of intrinsic functional connectivity MRI. *Nat. Neurosci.* 16 832–837. 10.1038/nn.3423 23799476

[B8] Cohen J. N. Drabick D. A. G. Blanco C. Schneier F. R. Liebowitz M. R. Heimberg R. G. (2017). Pharmacotherapy for social anxiety disorder: interpersonal predictors of outcome and the mediating role of the working alliance. *J. Anxiety Disord.* 52 79–87. 10.1016/j.janxdis.2017.10.004 29102818 PMC5689479

[B9] Dobrushina O. R. Vlasova R. M. Rumshiskaya A. D. Litvinova L. D. Mershina E. A. Sinitsyn V. E. (2020). Modulation of intrinsic brain connectivity by implicit electroencephalographic neurofeedback. *Front. Hum. Neurosci.* 14:192. 10.3389/fnhum.2020.00192 32655386 PMC7324903

[B10] Eller-Smith O. C. Nicol A. L. Christianson J. A. (2018). Potential mechanisms underlying centralized pain and emerging therapeutic interventions. *Front. Cell. Neurosci.* 12:35. 10.3389/fncel.2018.00035 29487504 PMC5816755

[B11] Fovet T. Micoulaud-Franchi J.-A. Vialatte F.-B. Lotte F. Daudet C. Batail J.-M. (2017). On assessing neurofeedback effects: should double-blind replace neurophysiological mechanisms? *Brain* 140:e63. 10.1093/brain/awx211 28969378

[B12] Friston K. (2010). The free-energy principle: a unified brain theory? *Nat. Rev. Neurosci.* 11 127–138. 10.1038/nrn2787 20068583

[B13] Gaul C. Brömstrup J. Fritsche G. Diener H. C. Katsarava Z. (2011). Evaluating integrated headache care: a one-year follow-up observational study in patients treated at the Essen headache centre. *BMC Neurol.* 11:124. 10.1186/1471-2377-11-124/FIGURES/3 PMC3203041 21985562

[B14] Green P. Macleod C. J. (2016). SIMR: an R package for power analysis of generalized linear mixed models by simulation. *Methods Ecol. Evol.* 7 493–498. 10.1111/2041-210X.12504

[B15] Gu Q. Hou J.-C. Fang X.-M. (2018). Mindfulness Meditation for Primary Headache Pain. *Chin. Med. J. (Engl).* 131 829–838. 10.4103/0366-6999.228242 29578127 PMC5887742

[B16] Harte S. E. Harris R. E. Clauw D. J. (2018). The neurobiology of central sensitization. *J. Appl. Biobehav. Res.* 23:e12137. 10.1111/jabr.12137

[B17] Hathaway S. R. McKinley J. C. (1943). *Mmpi. Manual for Administration and Scoring.* Minneapolis, MN: University of Minnesota Press.

[B18] Haufe S. DeGuzman P. Henin S. Arcaro M. Honey C. J. Hasson U. (2018). Elucidating relations between fMRI, ECoG, and EEG through a common natural stimulus. *Neuroimage* 179 79–91. 10.1016/j.neuroimage.2018.06.016 29902585 PMC6063527

[B19] Headache Classification Committee of the International Headache Society (IHS) (2013). The international classification of headache disorders, 3rd edition (beta version). *Cephalalgia* 33 629–808. 10.1177/0333102413485658 23771276

[B20] Hiltunen T. Kantola J. Abou Elseoud A. Lepola P. Suominen K. Starck T. (2014). Infra-slow EEG fluctuations are correlated with resting-state network dynamics in fMRI. *J. Neurosci.* 34 356–362. 10.1523/JNEUROSCI.0276-13.2014 24403137 PMC6608153

[B21] Ioannides A. A. (2018). Neurofeedback and the neural representation of self: lessons from awake state and sleep. *Front. Hum. Neurosci.* 12:142. 10.3389/fnhum.2018.00142 29755332 PMC5932408

[B22] Jensen R. Stovner L. J. (2008). Epidemiology and comorbidity of headache. *Lancet Neurol.* 7 354–361. 10.1016/S1474-4422(08)70062-0 18339350

[B23] Krupnick J. L. Sotsky S. M. Elkin I. Simmens S. Moyer J. Watkins J. (2006). The role of the therapeutic alliance in psychotherapy and pharmacotherapy outcome: findings in the national institute of mental health treatment of depression collaborative research program. *Focus* 4 269–277. 10.1176/FOC.4.2.269 8698947

[B24] Marzbani H. Marateb H. R. Mansourian M. (2016). Neurofeedback: a comprehensive review on system design, methodology and clinical applications. *Basic Clin. Neurosci.* 7 143–158. 10.15412/J.BCN.03070208 27303609 PMC4892319

[B25] Mathur S. Sutton J. (2017). Personalized medicine could transform healthcare. *Biomed. Rep.* 7 3–5. 10.3892/br.2017.922 28685051 PMC5492710

[B26] Melzack R. (1975). The McGill pain questionnaire: major properties and scoring methods. *Pain* 1 277–299. 10.1016/0304-3959(75)90044-5 1235985

[B27] Moshkani Farahani D. Tavallaie S. A. Ahmadi K. Fathi Ashtiani A. Sheikh M. Yahaghi E. (2014). Comparison of neurofeedback and transcutaneous electrical nerve stimulation efficacy on treatment of primary headaches: a randomized controlled clinical trial. *Iran. Red Crescent Med. J.* 16:e17799. 10.5812/ircmj.17799 25389484 PMC4222010

[B28] Napadow V. Kim J. Clauw D. J. Harris R. E. (2012). Brief Report: decreased intrinsic brain connectivity is associated with reduced clinical pain in fibromyalgia. *Arthritis Rheum.* 64 2398–2403. 10.1002/art.34412 22294427 PMC3349799

[B29] Napadow V. LaCount L. Park K. As-Sanie S. Clauw D. J. Harris R. E. (2010). Intrinsic brain connectivity in fibromyalgia is associated with chronic pain intensity. *Arthritis Rheum.* 62 2545–2555. 10.1002/art.27497 20506181 PMC2921024

[B30] Nestoriuc Y. Rief W. Martin A. (2008). Meta-analysis of biofeedback for tension-type headache: efficacy, specificity, and treatment moderators. *J. Consult. Clin. Psychol.* 76 379–396. 10.1037/0022-006X.76.3.379 18540732

[B31] Nicholson A. A. Ros T. Frewen P. A. Densmore M. Théberge J. Kluetsch R. C. (2016). Alpha oscillation neurofeedback modulates amygdala complex connectivity and arousal in posttraumatic stress disorder. *NeuroImage Clin.* 12 506–516. 10.1016/j.nicl.2016.07.006 27672554 PMC5030332

[B32] Othmer S. Othmer S. F. Kaiser D. A. Putman J. (2013). Endogenous neuromodulation at infralow frequencies. *Semin. Pediatr. Neurol.* 20 246–257. 10.1016/j.spen.2013.10.006 24365573

[B33] Othmer S. F. (2017). *Protocol Guide for Neurofeedback Clinicians*, 5th Edn. Los Angeles, CA: EEG Institute.

[B34] Ros T. Théberge J. Frewen P. A. Kluetsch R. Densmore M. Calhoun V. D. (2013). Mind over chatter: plastic up-regulation of the fMRI salience network directly after EEG neurofeedback. *Neuroimage* 65 324–335. 10.1016/j.neuroimage.2012.09.046 23022326 PMC5051955

[B35] Roy R. de la Vega R. Jensen M. P. Miró J. (2020). Neurofeedback for pain management: a systematic review. *Front. Neurosci.* 14:671. 10.3389/fnins.2020.00671 32765208 PMC7378966

[B36] Schabus M. Griessenberger H. Gnjezda M.-T. Heib D. P. J. Wislowska M. Hoedlmoser K. (2017). Better than sham? A double-blind placebo-controlled neurofeedback study in primary insomnia. *Brain* 140 1041–1052. 10.1093/brain/awx011 28335000 PMC5382955

[B37] Siniatchkin M. Hierundar A. Kropp P. Kuhnert R. Gerber W. D. Stephani U. (2000). Self-regulation of slow cortical potentials in children with migraine: an exploratory study. *Appl. Psychophysiol. Biofeedback* 25 13–32. 10.1023/A:1009581321624 10832507

[B38] Spielberger C. D. Gorsuch R. L. Lushene R. Vagg P. R. Jacobs G. A. (1983). *Manual for the State-Trait Anxiety Inventory.* Palo Alto, CA: Consulting Psychologists Press.

[B39] Stokes D. A. Lappin M. S. (2010). Neurofeedback and biofeedback with 37 migraineurs: a clinical outcome study. *Behav. Brain Funct.* 6:9. 10.1186/1744-9081-6-9 20205867 PMC2826281

[B40] Stovner L. Hagen K. Jensen R. Katsarava Z. Lipton R. Scher A. (2007). The global burden of headache: a documentation of headache prevalence and disability worldwide. *Cephalalgia* 27 193–210. 10.1111/j.1468-2982.2007.01288.x 17381554

[B41] Stovner L. J. Nichols E. Steiner T. J. Abd-Allah F. Abdelalim A. Al-Raddadi R. M. (2018). Global, regional, and national burden of migraine and tension-type headache, 1990–2016: a systematic analysis for the Global Burden of Disease Study 2016. *Lancet Neurol.* 17 954–976. 10.1016/S1474-4422(18)30322-3 30353868 PMC6191530

[B42] Strehl U. (2014). What learning theories can teach us in designing neurofeedback treatments. *Front. Hum. Neurosci.* 8:894. 10.3389/fnhum.2014.00894 25414659 PMC4222234

[B43] Strehl U. Aggensteiner P. Wachtlin D. Brandeis D. Albrecht B. Arana M. (2017). Neurofeedback of slow cortical potentials in children with attention-deficit/hyperactivity disorder: a multicenter randomized trial controlling for unspecific effects. *Front. Hum. Neurosci.* 11:135. 10.3389/fnhum.2017.00135 28408873 PMC5374218

[B44] Thorp S. L. Suchy T. Vadivelu N. Helander E. M. Urman R. D. Kaye A. D. (2018). Functional connectivity alterations: novel therapy and future implications in chronic pain management. *Pain Physician* 21 E207–E214. 29871376

[B45] Walker J. E. (2011). QEEG-guided neurofeedback for recurrent migraine headaches. *Clin. EEG Neurosci.* 42 59–61. 10.1177/155005941104200112 21309444

[B46] Wallasch T. M. Kropp P. (2012). Multidisciplinary integrated headache care: a prospective 12-month follow-up observational study. *J. Headache Pain* 13 521–529. 10.1007/S10194-012-0469-Y/FIGURES/1 22790281 PMC3444539

[B47] Witte M. Kober S. E. Wood G. (2018). Noisy but not placebo: defining metrics for effects of neurofeedback. *Brain* 141:e40. 10.1093/brain/awy060 29547965

[B48] Zilcha-Mano S. Roose S. P. Brown P. J. Rutherford B. R. (2019). Not just nonspecific factors: the roles of alliance and expectancy in treatment, and their neurobiological underpinnings. *Front. Behav. Neurosci.* 12:293. 10.3389/fnbeh.2018.00293 30760986 PMC6361734

